# Cultural Aspects of Social Anxiety Disorder: A Qualitative Analysis of Anxiety Experiences and Interpretation

**Published:** 2019-01

**Authors:** Abolafzl Mohammadi, Imaneh Abasi, Mehdi Soleimani, Seyed Tayeb Moradian, Taha Yahyavi, Mostafa Zarean

**Affiliations:** 1 Department of Psychiatry, School of Medicine, Tehran University of Medical Sciences, Tehran, Iran.; 2 Department of Clinical Psychology, University of Social Welfare and Rehabilitation Sciences, Tehran, Iran.; 3 Atherosclerosis Research Center, Baqiyatallah University of Medical Sciences, Tehran, Iran.; 4 Department of Psychiatry, School of Medicine, Tehran University of Medical Sciences, Tehran, Iran.; 5 Department of Psychology, School of Education and Psychology, Tabriz University, Tabriz, Iran.

**Keywords:** *Anxiety*, *Culture*, *Qualitative Research*, *Social Anxiety Disorder*

## Abstract

**Objective:** Anxiety is a complex phenomenon on which culture has a prominent influence. The present study aimed to investigate the cultural aspects of social anxiety disorder (SAD) in an Iranian population.

**Method** **:** A qualitative content analysis research was done to answer the study question. A total of 16 individuals with social anxiety disorder (six men and 10 women) were selected using purposeful sampling method (M = 24.43, SD = 4.56). The study was conducted in Tehran, Urmia, and Sanandaj- Iran. Participants were from different ethnic backgrounds (LOR, FARS, TURK, and KURD). Data were analyzed by thematic analysis using an inductive method.

**Results: **Analysis of participants’ records yielded five distinct categories with some subcategories, which are as follow: (1) anxiety experiences; (2) core beliefs; (3) reasons of being anxious; (4) effects of SAD on life aspects; and (5) coping strategies.

**Conclusion: **It seems that symptoms of social anxiety and its underlying beliefs, causes and effects and coping strategies are almost experienced and interpreted in a way that could be the same as DSM-5 clinical presentation of social anxiety, with the exception that somatic symptoms are experienced by almost all participants.

Anxiety disorders are the most prevalent psychiatric disorders (1). They affect various aspects of life, including social, financial, educational, relationship, and quality of life. Anxiety disorders are the result of genetic, environmental, familial, mental, and cultural factors (2, 3). Anxiety disorders are affected by cultural features (4, 5); and symptoms and variation in prevalence of anxiety disorders could be transformed across ethnicities and cultures (6, 7). 

Cultural values and beliefs may put individuals at risk and they may also act as a buffer against anxiety problems (8). Culture plays an important role in awareness toward problem severity and its consequences and health care searching behaviors (9). 

In line with cultural influences on anxiety disorders, a review showed that unexpectedness and 10-minute crescendo criteria in panic disorder, definition of social anxiety and social reference group in social anxiety disorder, and the preference given to psychological symptoms of worry in generalized anxiety disorder are phenomenological expressions in different cultures (10). Individualism vs. collectivism nature of countries may be related to social anxiety differently (11). Furthermore, khyâl cap (wind attacks), taijin kyofusho, and ataques de nervios are three main examples of culture-specific expressions of anxiety disorders, which have been included in DSM-V as distress concepts (12). 

A study on anxiety and its cultural complexities in Iran showed that individuals with Azeri ethnicity (one of several Iranian ethnicities) suffering from emotional disorders reported 11 main themes as avoidance, dysfunction, arousal, disorganized personality, repetition, somatization, problematic behavior, maladaptive cognition, awareness, and positive and negative emotionality, among which somatization had the highest frequency (13). There is strong evidence that individuals from non-Western cultures significantly report more somatic rather than psychological symptoms (14, 15). 

In this study, we used the term culture and ethnicity interchangeably to refer to common heritage, shared beliefs, and norms of a unit or a group of people. Iran is a country with different cultural and ethnical background, and this may be responsible for various psychological manifestations. Moreover, there is no adequate information about cultural aspects of anxiety in Iranian population and because of the prominence of cultural effects on anxiety symptoms (8, 9 and 12) and culture-gene interactions (16, 17), studying the cultural nature of anxiety disorders in Iranian population is of utmost importance. Furthermore, the anxiety phenomenon is extremely complex and passing of time makes it even more complex in some ways, so the best way to clarify a content-based phenomenon is to conduct a qualitative research.

The qualitative method can illuminate some phenomena which could not be reached out through qualitative methods. Moreover, it can also help understand different perspectives and contribute to mental health policy (18). The main objective of this research was to study the cultural aspects of anxiety, mainly social anxiety, in four ethnic groups (LOR, FARS, TURK, and KURD) in Iran, who were diagnosed with social anxiety disorder. Thus, generalization is possible. Specific aims in this research were as follow: describing anxiety experience among people suffering from anxiety disorders; evaluating awareness and experience of anxiety among general people; determining the causes of perceived anxiety among participants in the study; describing the participants’ opinions towards prognoses and consequences of anxiety, describing the participants’ opinions towards prevention and treatment of anxiety disorders.

## Materials and Methods

The study design is a qualitative content analysis research. Study conducted in the cities of Tehran, Urmia, and Sanandaj- Iran. A total of 16 individuals with social anxiety disorders (six men and 10 women) were selected via purposeful sampling method (M = 24.43, SD = 4.56). With respect to education level, nine participants had high school diploma, one had a bachelor’s degree, and six had a master’s degree. With respect to ethnicity, one participant was identified as Lor, two as Fars, six as Turk, and seven as Kurd. Patients were selected according to some inclusion and exclusion criteria and were recruited from counseling centers in aforementioned cities. Main inclusion criteria were age>18 and primary diagnosis of social anxiety disorder. Main exclusion criteria were as follow: comorbid with any debilitating mental illness like schizophrenia, bipolar disorder, substance abuse, and mental retardation; comorbid with debilitating physical illness. The study was done during 2016-2017. All individuals were interviewed by a psychiatrist and were diagnosed as social anxiety disorder. Then, they were informed about the purpose of study, and informed consent was obtained from all of them. All voluntary participants were asked open ended questions by a clinical psychologist (MS.c.) through an in-depth and semi-structured interview. Answers were audio recorded and lasted 30 to 60 minutes. Questions were categorized into three main groups: (1) personal experiences and perception about anxiety; (2) factors causing anxiety; and (3) coping strategies when feeling anxious. Ethical approval was obtained from the National Institute for Medical Research Development. Informed consent was obtained before interviewing. Participants were assured about the confidentiality of their information and were debriefed about the purpose of the study. 

***Analysis***

Results of the interview with each participant were recorded, coded, and categorized. Overall, the interviewed data were processed through thematic analysis. An inductive method was used for data analysis (19).

## Results

Participants’ description of their experiences and beliefs about coping consequences of anxiety, SAD reasons, and coping strategies when facing anxiety were categorized in the following categories (Table 1).

**1** **.** **Symptoms of Anxiety**

1.1 Emotional

Anxiety was experienced mainly in public (feeling anxious when speaking, asking questions, and commenting) and in classroom (anxiety when presenting, providing training courses, asking questions, speaking, and commenting). 

Example

Participant 1: I am afraid of speaking in public because I feel I am not good at it. 

1.2. Cognitive 

Cognitive aspect of anxiety was categorized into two main categories as pre event rumination (thinking of making a mistake and thinking of being humiliated, thinking of being mocked, and thinking of not being able to handle the situation) and post event rumination (thinking of making mistakes, thinking of being humiliated, and thinking of being mocked). 

Example

Participant 2. I am afraid of making mistakes and classmates laugh at me because of it. 

1.3 Behavioral

Behavioral aspect was categorized into 3 main sections as avoidance (avoiding stressful situations), surrender (inability of movement and inability to speak), and safety behaviors (talking fast). 

Example

Participant 5: When I am in stressful situations I can’t speak or move, it is like I am in a cage. 

1.4 Physical

Physical aspect consisted of several symptoms including increasing heartbeat, feeling cold, feeling pressure on the head, sweating, hoarseness, inability to swallow saliva, suffocation, body weakness, lowering the pressure of the body, blushing, feeling hot in the body, difficulty sleeping, feeling of losing balance, flushing, and lowering the tone of voice. 

Example

Participant 9: when I am anxious, I feel flushed, I feel I am losing balance, and I feel hot in my body.

**2** **.** **Core Beliefs**

Profound beliefs related to social anxiety experienced by participants were categorized in following categories:

2.1 Fear of negative evaluation: Being neglected by others, rejected, mocked by others, loss of pride, loss of popularity, and feeling ashamed.

Example 

Participant 10: When I want to give presentation in class, I feel like everybody is looking at me, they are making fun of me, and they think I am not good at it. 

2.2 Unworthiness: Not being worthy, not being satisfied with oneself. 

Example 

Participant 12: I think something makes me most vulnerable in these situations and that is I don’t feel worthy enough.

2.3 Incompetency: loss, failure.

Example

Participant 7: When I think of my SAD symptoms, I think about my previous failures.

**3** **.** **Reasons of Being Anxious**

3.1 Familial factors: Family tension, parental punishment, parental harshness, incorrect parenting systems, and emotional deprivation.

Example

Participant 13: I was raised in a family full of tension and stress that were beyond my ability to manage. 

3.2 Fear: Fear of negative evaluation, fear of being rejected, and losing loved ones. 

Example

Participant 13: I think fear of other`s views about us and fear of being rejected by them can make us anxious.

3.3 Core beliefs: Unworthiness and incompetency.

Example 

Participant 16: In an anxious situation, I really feel like I can’t help myself and this may be the reason of my anxiety.

3.3 Society: Poverty and school (poor education and inappropriate laws). 

Example

Participant 15: I have been faced with poverty and other stresses several times and since then I have been so tensed and anxious.

**4** **.** **Effects of SAD on Life Aspects**

4.1. Social dysfunction (inability to speak in front of others, inability to connect with others, inability to make friends, inability to go out alone, losing job opportunities, avoiding social relationships, and failure to establish a relationship with the opposite sex).

4.2. Emotional dysfunction (worry, lowering fear threshold, and feeling disappointed)

Example 

Participant 14: Social anxiety has ruined my life. I am always worried about social situations. 

4.3. Behavioral Dysfunction (poor quality of life, avoiding stressful situations)

Example 

Participant 11: I don’t take part in social situations where I am not acquainted.

**5** **.** **Coping Mechanisms**

Participant’s explanation about strategies for handling their anxiety was categorized in following sections:

5.1. Treatment (psychotherapy and pharmacotherapy)

Example

Participant 12: A psychologist or a psychiatrist may be helpful. 

5.2. Emotion-based mechanisms (watching movies, overeating, sleeping, avoiding stressful situations, praying, talking to others, drinking, smoking, listening to music, and relaxing)

Example 

Participant 11: When I am worried or anxious, I eat and sometimes I listen to music.

5.3. Problem-based strategies (studying psychological books, practicing for presentation or speaking, studying more about the presentation subject).

Example

Participant 8: I analyze the situation and find some logical solutions for it, but it doesn’t always work. 

5.4. Behavioral and Cognitive Strategies (positive self-statements, distractions, and avoiding eye contact).

Example

Participant 5: In classroom, when I am talking about something, I cannot look in the eyes of others. 

Furthermore, when participants were asked about the onset of their social anxiety symptoms, some of them stated that they had these feelings since childhood and most of them pointed to some events, such as a new job/changing job, teachers, and inappropriate behavior.

**Table 1:** Participants’ Categories of Experienced Social Anxiety Symptoms Presented as Main Categories, Subcategories, and Meaningful Codes

**Categories**	**Subcategories**	**Codes**
Anxiety experiences	Emotional (public and classroom)	Feeling anxious when speaking Asking questions Commenting Providing training courses
	Cognitive (pre event rumination, and post event rumination)	Thinking of making mistakes Thinking of being humiliated Thinking of being mocked Thinking of not being able to handle the situation
	Behavioral (avoidance, surrender, and safety behaviors)	Avoiding stressful situations Inability to move and inability to speak Talking fast
	Physical	Increasing heartbeat Feeling cold in the body Feeling pressure on the head Sweating Hoarseness Inability to swallow saliva Suffocation Body weakness Lowering the pressure of the body Blushing Feeling hot in the body Difficulty sleeping Feeling losing balance Flushing Lowering the tone of voice
Core beliefs	Fear of negative evaluation	Being neglected by others Being rejected Bg mocked by others Loss of pride Loss of popularity Feeling ashamed in front of others The negative thoughts of others about oneself
	Unworthiness	Not being worthy Not being satisfied with oneself
	Incompetency	Loss, failure
Reasons of being anxious	Familial factors	Family tension Parental punishment Parental harshness Incorrect parenting systems Emotional deprivation
	Fear	Fear of negative evaluation Fear of being rejected Fear of losing important others
	Core Beliefs	Unworthiness and incompetency
	Society	Poverty and school
Effects of SAD on life aspects	Social dysfunction	Inability to speak in front of others, inability to connect with others, inability to make friends, inability to go out alone, losing job opportunities, avoiding social relationships, little to say, and failure to establish a relationship with the opposite sex
	Emotional dysfunction	Worry Lowering fear threshold Feeling disappointed
	Behavioral dysfunction	Poor quality of life, avoiding stressful situations
Coping mechanisms	Treatment	Psychotherapy Pharmacotherapy
	Emotion-based mechanisms	Watching movies Overeating, sleeping Avoiding stressful situations Praying Talking with others Drinking Smoking Listening to music Relaxation
	Problem-based mechanisms	Studying psychological books to practice for presentation or speaking Studying more about the presentation subject
	Behavioral and Cognitive strategies	Positive self-statements Distractions Avoiding eye contact

## Discussion

The present research was a qualitative study on anxiety experiences and responses to anxiety among individuals suffering from social anxiety disorder. This was the first study in Iran to investigate some questions surrounding the underlying experiences of anxiety and coping strategies toward anxiety. Analysis of participants’ records yielded five distinct themes: (1) anxiety experiences; (2) core beliefs; (3) reasons of being anxious; (4) effects of SAD on life aspects; and (5) coping strategies. 

Anxiety symptoms reported by participants in this study are similar to the last DSM criteria on SAD symptoms showing that these symptoms are being experienced globally (20). Moreover, these findings are comparable with previous studies showing that there are no significant differences between Americans and Japanese on SAD symptoms; however, Taijin Kyofusho, which is a form of social anxiety in Japanese culture, was more reported by Japanese individuals (21), indicating that some form of SAD may be more culturally dependent and treatment should be tailored for them.

Physical symptoms, not a main symptom of SAD, were reported significantly and by most participants of the present study. This finding is consistent with that of previous studies demonstrating this notion that people in non-Western or Eastern countries (9), including Iran (22), tend to somatize their distress and anxiety because of different ethnomedical beliefs about physical symptoms (23) or because they have learned to somatize their distress to get more attention. 

Results of core beliefs showed 3 main subthemes as fear of negative evaluation, unworthiness, and inadequacy. Fear of negative evaluation is the defining feature and core of social anxiety (20). Unworthiness and inadequacy are two underlying cognitions in classical cognitive theory that predispose individuals to interpret stimuli in a distorted way and as a result experience social anxiety symptoms (24). 

In case of causes of SAD, participants attribute SAD to psychological (fear and core beliefs) and non-psychological (society and family) processes; and the impact of non-psychological process was highlighted. This is in line with a previous study (25) and indicate that Iran as a developing country with a different culture and various ethnicities is exposed to many social, economic, and political deprivations, which affect Iranians’ life in unpleasant ways. 

Analysis of data revealed that social anxiety symptoms affect individuals’ life in various ways (social, emotional, and behavioral), and this supports previous studies indicating low quality of life in the participants (26).

With respect to coping strategies, four main themes (treatment, emotion-based mechanisms, problem-based mechanisms, and behavioral and cognitive strategies) were reported. Among them, praying and a special bond with God were significantly reported. This finding showed that Iran is a religious country and spirituality could make a bigger difference in people’s lives and it is used willingly by individuals with SAD as one of main coping strategies. Inconsistent with this result, previous studies have indicated the contributory role of religion and spirituality in SAD (27).

## Limitation

This study was the first in its field of inquiry. However, there were some limitations that should be considered carefully. First, this was a qualitative study and thus findings should be generalized to other SAD individuals with caution. Second, although in the present study participants were from different ethnicities, the difference between ethnicities in experiencing anxiety and its related factors was not investigated. Thus, future studies should be conducted to take a deeper look into this topic. Third, there are some transdiagnostic and common factors between disorders, especially emotional disorders, which play an important role in etiology and treatment of emotional disorders. Finally, the present study assessed only four ethnicities and did not include others.

## Conclusion

The present study highlighted the importance of experiencing social anxiety, interpretation of causes of social anxiety, underlying beliefs of SAD and its effects, and coping strategies in the diverse ethnic population of Iran. Moreover, this study was the first of its kind, especially in Iran, as a Middle-Eastern country. Findings of the present study indicated that SAD symptoms and the related psychological processes are a global problem and this could be due to social media and other mass media making people around the world more and more alike.
